# Adapting the randomised controlled trial (RCT) for precision medicine: introducing the nested-precision RCT (npRCT)

**DOI:** 10.1186/s13063-020-04965-0

**Published:** 2021-01-06

**Authors:** Nils Kappelmann, Bertram Müller-Myhsok, Johannes Kopf-Beck

**Affiliations:** 1 Department of Translational Research in Psychiatry, Max Planck Institute of Psychiatry, Kraepelinstraße 2-10, 80804 Munich, Germany; 2 International Max Planck Research School for Translational Psychiatry (IMPRS-TP), Munich, Germany; 3 Department of Health Data Science, University of Liverpool, Liverpool, UK; 4 Max Planck Institute of Psychiatry, Munich, Germany

**Keywords:** Randomised controlled trial, Nested-precision randomised controlled trial, Precision medicine

## Abstract

Adaptations to the gold standard randomised controlled trial (RCT) have been introduced to decrease trial costs and avoid high sample sizes. To facilitate development of precision medicine algorithms that aim to optimise treatment allocation for individual patients, we propose a new RCT adaptation termed the nested-precision RCT (npRCT). The npRCT combines a *traditional* RCT (intervention A versus B) with a *precision* RCT (stratified versus randomised allocation to A or B). This combination allows online development of a precision algorithm, thus providing an integrated platform for algorithm development and its testing. Moreover, as both the *traditional* and the *precision* RCT include participants *randomised* to interventions of interest, data from these participants can be jointly analysed to determine the comparative effectiveness of intervention A versus B, thus increasing statistical power. We quantify savings of the npRCT compared to two independent RCTs by highlighting sample size requirements for different target effect sizes and by introducing an open-source power calculation app. We describe important practical considerations such as blinding issues and potential biases that need to be considered when designing an npRCT. We also highlight limitations and research contexts that are less suited for an npRCT. In conclusion, we introduce the npRCT as a novel precision medicine trial design strategy which may provide one opportunity to efficiently combine traditional and precision RCTs.

## Background

In the wake of precision medicine, adaptations of the “gold standard” randomised controlled trial (RCT) have been introduced to accelerate the identification of personalised treatments, decrease trial costs, and avoid unnecessarily high sample sizes. These include adaptation studies such as the platform, linked Bayesian adaptive randomisation, or leapfrog design that utilise early stopping rules or (biomarker-dependent) mid-trial changes in allocation ratio [1–4]. We propose a new RCT adaptation that could facilitate the move towards precision medicine, which aspires to give personalised treatment suggestions based on participant characteristics or pathological markers.

### The problem of retrospective precision algorithm development

To move towards precision medicine, researchers need to identify participant characteristics or pathological markers that are prescriptive, meaning they are informative on whether any individual participant benefits more from one treatment over another. As prescriptive markers for different interventions are often not known, current research efforts often revolve around re-analysing existing RCT data to identify pre-treatment prescriptive variables and define precision algorithms.

A promising example for precision algorithm development in psychiatry is the Personalised Advantage Index (PAI) [5]. The PAI was developed based on a combination of prescriptive pre-treatment variables and provides a quantitative preference score on whether intervention A or intervention B is likely to be the optimal treatment for any individual participant. While approaches such as the PAI are fruitful for the development of new stratification rules, previous efforts have largely been restricted to algorithm development using existing RCT data. If precision algorithms were to find their way into clinical care, however, they would ideally require prospective testing similar to how interventions are evaluated more generally; that is, while interventions need to show empirical superiority over placebo or current gold standard treatments, precision algorithms should exhibit empirical superiority over random allocation to interventions.

To address the problematic predominance of retrospective precision algorithm development and lack of prospective evaluation, we propose what we term nested-precision RCT (npRCT), which allows for integrated precision algorithm development and evaluation.

## Introducing the npRCT

### Design and statistical power

**Fig. 1:**
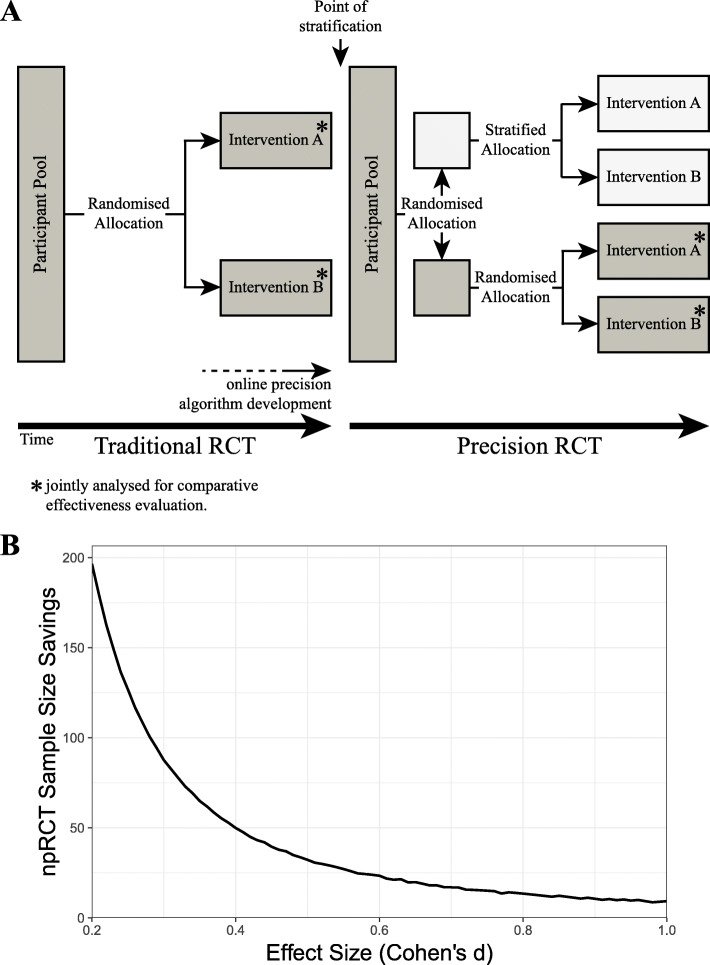
**a** The npRCT design. **b** Sample size savings of the npRCT compared to two independent RCTs is described for an example comparison of two interventions on a continuous outcome and with varying effect sizes (as Cohen's *d*)

As depicted in Fig. 1a, the npRCT combines a *traditional* RCT, comparing intervention A versus B, with a *precision* RCT, comparing stratification to intervention A or B versus randomisation to A or B.

This RCT combination has a twofold purpose. First and as noted above, combining these two RCT types can provide an integrated platform that allows for sequential development and testing of a precision algorithm. Specifically, researchers can analyse collected data during the *traditional* RCT to see if any strong prescriptive variables are present that could guide treatment choice. For example, identification of pre-treatment prescriptive variables can be conducted using the PAI approach, which looks for treatment-moderating variables using different regression-based and machine learning techniques [6]. Ideally, any of such online analyses of traditional RCT data should be conducted by an independent trial collaborator/statistician that is not involved in day-to-day recruitment or treatment procedures, so as to prevent potential researcher biases. If prescriptive variables are identified that could optimise intervention allocation decisions, researchers can move on to the *precision* RCT stage, in which the algorithm is prospectively evaluated against random intervention allocation.

The second purpose of the npRCT is to offer a design that can save trial costs as compared to running traditional and precision RCTs independently. As both the traditional and the precision RCT include participants randomised to interventions of interest, the traditional RCT can be understood as *nested* within the combined npRCT design. As such, all individuals randomised to interventions of interest can be jointly analysed to determine the comparative intervention effectiveness of interventions A and B. This nesting increases statistical power by reducing the sample size of the *traditional* RCT by 1/2 times the sample size of the *precision* RCT.

### Practical considerations

We want to highlight three major practical considerations that should be made when setting up an npRCT.

First, researchers should consider effect size and allocation ratio criteria a priori that determine if it is sensible to move on to the precision RCT stage or to maintain the traditional RCT. In terms of effect sizes, researchers need to specify minimum effect size benefits of the precision algorithm. If an effective algorithm can be identified based on this criterion, researchers can move on to the precision RCT stage. If an effective algorithm cannot be identified based on this criterion, researchers could maintain the traditional RCT design to focus on the comparative intervention effectiveness evaluation. Similarly, researchers should carefully consider how overall intervention main effects are weighed against treatment-moderating effects: Take a hypothetical example where intervention A is more effective than intervention B overall (i.e. a significant intervention main effect is present). Under this scenario, the algorithm may allocate a majority of participants to intervention A and only few participants to intervention B. If effectiveness differences are too large, a precision algorithm will lose its economic value as a precision medicine tool as it would make more sense to allocate all participants to intervention A irrespective of any prescriptive variables.

Second, researchers need to carefully consider potential blinding issues and biases arising from the npRCT, which are particularly likely if interventions cannot be concealed (e.g. through use of matched drug capsules). For example, researchers might learn how the precision algorithm allocates participants to interventions, which could lead to potential experimenter biases. Experimenter and expectation biases may also change from traditional to precision RCT stages. For example, participants might have a clear expectation that intervention A works best for them, so may assume (if allocated to intervention B) in the precision RCT stage that they were not assigned by the precision algorithm. To avoid expectation biases at the stratification stage, the aim should be that all individuals involved in the trial (i.e. participants, clinicians, and the research team) are blinded, so that knowledge about allocation group will not affect trial results.

Third, it will be important to set up a timeline that leaves sufficient time for online precision algorithm development. It is sensible to start precision algorithm development relatively late in the traditional RCT stage, so as to maximise sample sizes used for algorithm identification. However, the benefit of maximising sample sizes for algorithm identification should be weighed against practical time requirements to conduct analyses, especially if recruitment rate is high, to avoid potential delays between traditional and precision RCTs at the point of stratification.

### Power calculation app

We have developed a free, open-source power calculation app for the npRCT that can be accessed via https://nprct.shinyapps.io/nprct/. This app includes power calculation strategies for (i) comparison of two groups on a continuous outcome (based on two-sample independent *t* tests), (ii) comparison of three or more groups on a continuous outcome (based on analysis of variance or ANOVA), and (iii) comparison of two or more groups on a binary or categorical outcome (based on *χ*^2^ tests).

This power calculation app shows savings with this design (in terms of participants per group) compared to running the traditional and the precision RCT independently (see Fig. 1b). The power calculation app also provides the transition point from *traditional* to *precision* RCT (cf. point of stratification); that is, the point of recruitment at which one needs to change towards the precision RCT design. Considering a continuous outcome and comparison of two interventions, for frequently reported small, medium, and large effect sizes, the npRCT would require 196 (Cohen’s *d* = 0.2), 32 (*d* = 0.5), and 13 (*d* = 0.8) participants fewer *per group* compared to two independent RCTs (assuming α = 0.05 and power = 0.8). These statistics emphasise recruitment savings gained by combining/nesting RCTs under the npRCT design.

In the power calculation app, we provide explanations on further practical and ethical considerations. We also provide a walk-through research example for the npRCT. Source code of the npRCT app and power computation functions are freely available via github under https://github.molgen.mpg.de/mpip/npRCT.app, which we encourage researchers to use and adapt for more complex npRCT design setups (e.g. with traditional RCT allocation ratios deviating from 1:1 allocation).

## Advantages and limitations of the npRCT

We have proposed the npRCT as a strategy to integrate precision algorithm development and testing of stratified versus randomised allocation in a unified design and outlined potential advantages of this design in terms of participant savings. Despite these advantages, there are several limitations of the npRCT that researchers need to consider when deciding between clinical trial designs.

First, the npRCT does not allow answering of all research questions relevant for precision medicine. For instance, the precision RCT stage may enable investigators to obtain a “theoretical” estimate of precision algorithm effectiveness compared to random treatment allocation assuming that the biases mentioned under the Practical considerations section are minimised. In clinical practice, however, stratification algorithms may not be used as absolute decision rules to select between treatments. Instead, algorithmic decisions may be used as support decision-making tools to guide clinicians’ and patients’ choices, so patients and clinicians could opt to ignore the algorithmic recommendation as well. To obtain a “practical” estimate of precision algorithm effectiveness as a support decision-making tool would thus instead require an evaluation of randomised treatment allocation versus algorithmic treatment recommendation, which may not be a suitable research question for the npRCT due to unique blinding and bias considerations.

Second, the assumption of ethical favourability of the npRCT needs to be assessed individually for each research context. For instance, recent simulation work on precision algorithm development for major depression has suggested that precision algorithm development itself requires very large sample sizes for a range of plausible algorithmic effect sizes reported in the literature [7]. In this report, the authors have even suggested that algorithm development might realistically benefit from development in non-randomised study settings, only to be evaluated in a randomised trial subsequently. If npRCT power calculation proves unrealistic for a specific research context, we recommend the use of alternative trial designs including separate algorithm development and testing.

## Conclusion

We describe the npRCT, which combines two RCTs addressing research questions on (i) comparative intervention effectiveness (intervention A versus B) and (ii) precision treatment allocation (stratified allocation versus randomised allocation to A or B). Combining these two designs allows integrated precision algorithm development and testing. It also increases statistical power, which can be quantified using our free, open-source power computation app. Based on the resulting participant savings, the npRCT may be ethically and economically favourable to conducting two RCTs independently for certain research contexts. As such, we hope the npRCT may constitute one means of fostering prospective testing of precision medicine algorithms.

## Data Availability

As described in the manuscript, we provide a free, open-source power calculation app for the npRCT design under https://nprct.shinyapps.io/nprct/. Code for the app and npRCT power calculation functions can be retrieved from https://github.molgen.mpg.de/mpip/npRCT.app.
